# Image‐domain metal artifact reduction in low‐energy VMI using high‐energy regional prior

**DOI:** 10.1002/mp.18118

**Published:** 2025-09-12

**Authors:** Dan Wang, Yu Zou, Qilin Zhang, Yi Yang, Zhe Shi, Juying Huang, Zhi Yang

**Affiliations:** ^1^ School of Biomedical Engineering Capital Medical University Beijing China; ^2^ Laboratory for Clinical Medicine Capital Medical University Beijing China; ^3^ Beijing Key Laboratory of Fundamental Research on Biomechanics in Clinical Application Capital Medical University Beijing China; ^4^ Medichord LLC Lewes Delaware USA; ^5^ Independent consultant Shenyang Liaoning China

**Keywords:** low energy spectral imaging, metal artifact reduction, virtual monochromatic imaging

## Abstract

**Background:**

Metal artifacts degrade the clinical utility of virtual monochromatic images (VMIs), particularly in low energy levels. Nevertheless, low‐energy VMIs have essential clinical applications, such as reducing the volume of iodinated contrast material administered, salvaging poorly attenuated contrast‐enhanced CT studies, and analyzing arterial vasculature during the venous phase. Conventional metal artifact reduction algorithms may introduce new artifacts and obscure soft tissue details.

**Purpose:**

The aim of this study is to develop a practical image‐domain solution for significantly reducing the metal artifacts in low‐energy VMIs while preserving the clarity of soft tissues and metal boundaries.

**Methods:**

A mapping model was developed to establish a relationship between optimal VMI and the material basis images (MBIs) in artifact‐free regions. This model was subsequently used to correct artifact‐affected regions in MBIs. Finally, artifact‐reduced low‐energy VMIs were synthesized from the updated MBIs. The approach, referred to as regional model‐based metal artifact reduction (rMAR), utilized the mapping model to effectively reduce metal artifacts. To validate the efficacy of the proposed method, both phantom and patient data acquired from Philips scanner were used. The scanner's built‐in metal artifact reduction for orthopedic implants, known as OMAR, was employed. Comprehensive comparisons were conducted among four image processing strategies: VMI alone, VMI combined with OMAR (VMI + OMAR), VMI combined with the proposed rMAR (VMI + rMAR), and a combination of all three methods (VMI + OMAR + rMAR). Evaluations were performed using visual assessment, line profile analysis, and measurement of the ∆CT number.

**Results:**

High‐energy VMIs exhibit significantly fewer metal artifacts compared to those at low energy levels, as demonstrated in both phantom and patient results. Although conventional metal artifact reduction algorithms can mitigate the existing artifacts, they often introduce new ones. In contrast, the proposed rMAR method effectively reduces artifacts in low‐energy VMIs, achieving improved image quality without introducing new artifacts. In specific cases, such as postoperative VMIs of hip prosthesis implants, the combined VMI + OMAR + rMAR approach demonstrates superior metal artifact reduction compared to either OMAR or rMAR alone. Quantitative line profile analysis indicated that the proposed rMAR method produced images with artifact levels more closely resembling the ground truth than those processed with OMAR. The ΔCT number was significantly lower in the images processed with rMAR than in those processed with OMAR.

**Conclusion:**

The proposed rMAR method effectively achieves metal artifact reduction, particularly in low‐energy VMIs, while preserving the clarity of soft tissues and metal boundaries. Consequently, the diagnostic value of low‐energy VMIs containing metal implants is enhanced.

## 1 INTRODUCTION

Metal artifacts frequently occur in clinical imaging when metal implants are present, such as hip prostheses, dental implants,^1^ metal clips,^2^ and pedicle screws.^3^ These artifacts result from beam hardening, scattering, or photon starvation, significantly degrading image quality and diagnostic accuracy.

Various methods have been developed to reduce metal artifacts, which can be broadly categorized into interpolation‐based metal artifact reduction (MAR) algorithms,^4^ spectral CT, and deep learning.^5^, ^6^, ^7^ However, deep learning methods require a substantial amount of training data.^8^ Classic metal artifact reduction methods include normalized metal artifact reduction (NMAR)^9^ and frequency split metal artifact reduction (fsMAR).^4^ These classic methods typically include key steps such as forward projection, segmentation, and interpolation.^4^, ^9^, ^10^ MAR algorithms can be applied in either the projection domain or image domain. However, their performance is sensitive to the shape, material, and size of metal implants. Irregular implant geometries (such as pedicle screws) or complex surrounding tissue structures may lead to intricate artifacts.^11^ To further improve image quality, iterative algorithms are often combined with MAR.^11^, ^12^, ^13^, ^14^, ^15^ Iterative metal artifact reduction (iMAR, Siemens Healthcare) integrates both fsMAR and NMAR.^11^ Metal artifact reduction for orthopedic implants (OMAR, Philips Healthcare) is an iterative projection‐based modification technique.^16^ Smart MAR (sMAR, GE Healthcare) also uses an iterative method, combining projection‐based and image‐based processing techniques.^17^

The types of spectral CT include dual‐source CT, dual‐layer CT, rapid kVp‐switching CT, single‐source dual‐scan CT, and split‐filter CT.^18^, ^19^, ^20^ Particularly, the effectiveness of VMIs in reducing metal artifacts is highly dependent on the selected energy.^21^ Numerous studies have demonstrated that high‐energy VMIs are more effective than low‐energy VMIs in reducing metal artifacts.^16^, ^22^ Studies have indicated that the optimal energy range for imaging pedicle screws is around 100–120 keV.^3^ For the hip implants, the optimal energy range is between 100 and 130 keV.^5^ To improve image quality, VMIs are usually combined with MAR techniques in commercial spectral CT systems.^18^

In summary, high‐energy VMIs are mainly used for metal artifact reduction, whereas low‐energy VMIs are applied to enhance soft tissue contrast. Clinically, low‐energy VMIs play a key role in reducing the dose of iodinated contrast media, salvaging poorly contrast‐enhanced CT scans, and analyzing arterial vasculature during the venous phase.^22^ However, in the presence of metal implants, low‐energy VMIs are prone to serious artifacts that can compromise their diagnostic value. Although the MAR algorithm and VMIs are usually combined, they can introduce new artifacts. Therefore, developing artifact‐suppressed low‐energy VMIs would be of high clinical significance.

To address this issue, we propose a model‐based algorithm that significantly reduces metal artifacts in low‐energy VMIs, assuming that a high‐energy VMI with fewer artifacts is available. To the best of our knowledge, this is the first time such an algorithm has been introduced. The proposed method operates in the image domain, making it compatible with various spectral CT imaging systems and enhancing the diagnostic utility of low‐energy VMIs.

## 2 MATERIALS AND METHODS

### 2.1 Imaging protocol

#### 2.1.1 Phantom data

**FIGURE 1:**
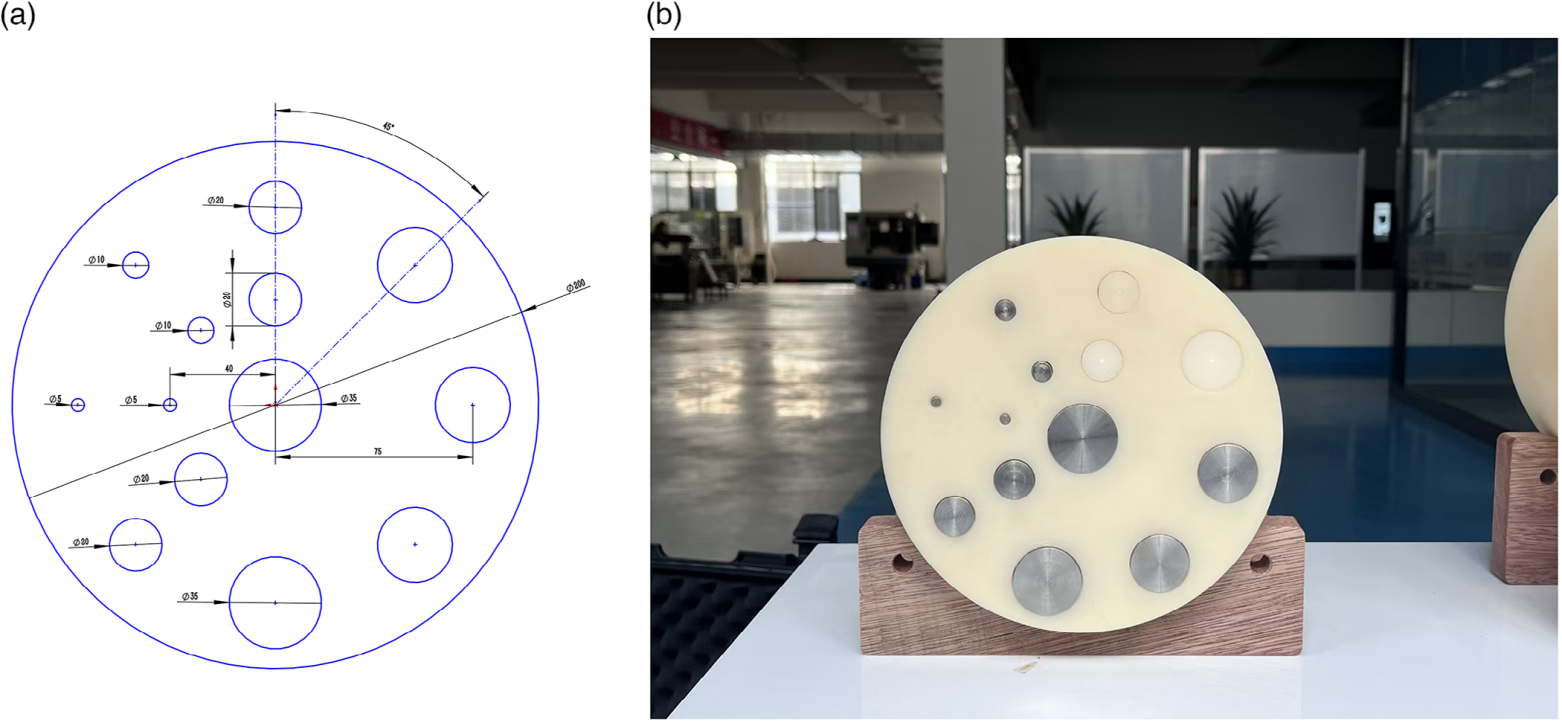
Test phantom. (a) Sketch of the custom‐designed test phantom. (b) Actual phantom.

A custom‐designed cylindrical phantom (200 mm in diameter) made of solid water was used in this study. The phantom contains multiple circular voids with diameters of 5, 10, 20, 28.5, and 35 mm, distributed as shown in Figure 1(a). In this study, the phantom is embedded with two titanium rods with a diameter of 10 mm each, as shown in Figure 1(b). The phantom was scanned using a high‐end spectral CT system (IQon, Philips Healthcare). The ground truth is the corresponding VMI with all water insertions. The scanner's built‐in MAR for orthopedic implants, called OMAR,^16^ is an iterative method optimized for imaging orthopedic devices. During data collection, the phantom was placed at the geometric isocenter of the scanner. Due to the detector configuration, data were acquired at 140 kV. The system uses dual‐layer detectors with a single x‐ray tube: the superficial (inner) layer, composed of yttrium‐based material, captures lower‐energy photons, while higher‐energy photons pass through to the deeper layer.^20^ Spectral reconstructions are available when imaging is performed at 120 or 140 kV. The field of view (FOV) for the phantom was 350 mm. The reconstructed image has a slice thickness of 2 mm and a matrix size of 512×512. The CTDIvol was 12 mGy. The window level/width of 0/200 HU was used for image display.

#### 2.1.2 Patient data

To evaluate the robustness of the proposed algorithm, clinical CT data from three patients with metal implants were retrospectively analyzed. The cases included: (1) a patient with pedicle screws, (2) a patient with dental fillings, and (3) a patient with a hip prosthesis. These cases were designated as patient 1 to patient 3. VMIs were reconstructed both with and without the application of OMAR techniques. All images were reconstructed with 2‐mm slices and a 512×512 matrix. iDose is an iteration algorithm with levels from 0 (off) to 5 (maximum). Detailed imaging parameters are summarized in Table 1. The study was ethically approved by the Ethical Committee of Capital Medical University, Beijing, China (No. Z2024SY064). Written consent from the participants was waived due to the retrospective design of the study.

**TABLE 1:** Patient data.

Patient index	Gender	Protocol	Site	Metal type	CTDIvol (mGy)	iDose level (0–5)
1	Female	Helical	Vertebra	Pedicle screw	8.6	4
2	Male	Helical	Head	Dental fillings	27	3
3	Female	Helical	Hip	Hip implants	15	1

### 2.2 VMIs reconstruction

In spectral imaging, the relationship between VMI at a given energy and the material basis images (MBIs) can be expressed mathematically as follows:

(1) $$\begin{array}{c} \left\{\begin{array}{c} f_{vmi}\;(E_{1})=\frac{1000}{\rho_{w}}\;\times \left[\left(\frac{m_{1}\left(E_{1}\right)}{m_{w}\left(E_{1}\right)}\right)\times f_{MBI1}+\left(\frac{m_{2}\left(E_{1}\right)}{m_{w}\left(E_{1}\right)}\right)\times f_{MBI2}\right]-1000\\ f_{vmi}\;(E_{2})=\frac{1000}{\rho_{w}}\;\times \left[\left(\frac{m_{1}\left(E_{2}\right)}{m_{w}\left(E_{2}\right)}\right)\times f_{MBI1}+\left(\frac{m_{2}\left(E_{2}\right)}{m_{w}\left(E_{2}\right)}\right)\times f_{MBI2}\right]-1000 \end{array}\right. \end{array}$$

where $m_{1}\left(E\right)$, $m_{2}\left(E\right)$, and $m_{w}\left(E\right)$ represent the mass attenuation coefficients of material 1, material 2, and water at energy E, respectively. $\rho_{w}$ denotes the water density. $f_{vmi}\;(E)$ is the VMI at energy E. $f_{MBI1}$ and $f_{MBI2}$ denote the MBIs of material 1 and material 2, respectively.

Based on the equations above, MBIs can be calculated from VMIs at two different energy levels in the image domain. If VMIs contain residual artifacts, these artifacts will propagate to MBIs. Conversely, if the MBIs are free from artifacts, the synthesized VMIs, as well as related outputs such as density images and effective atomic number images, will also be corrected accordingly.

### 2.3 Metal artifact reduction for low‐energy VMIs

**FIGURE 2:**
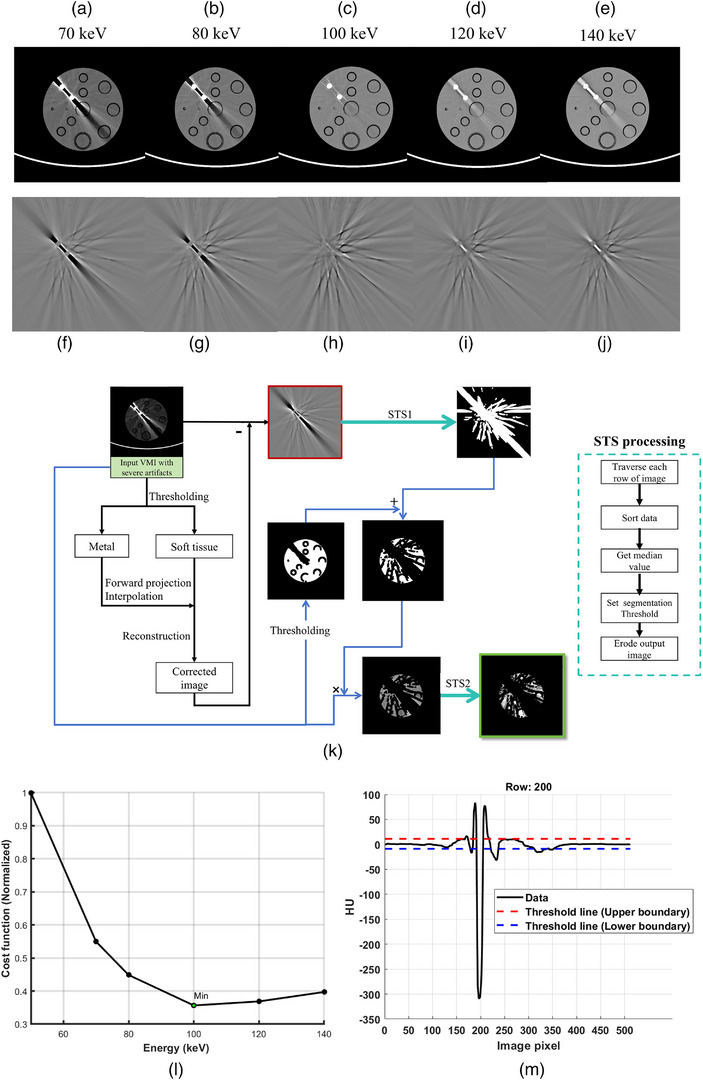
(a–e) is the VMIs sequence. The phantom consists of a water phantom (200 mm) and two Ti rods (10 mm). Artifact images (f–j) were extracted from (a–e). (k) Scheme of obtaining artifact masks and STS processing workflow. (l) The cost function curve for (f–j). The window level and width were set to 0/200 HU. (m) A case of STS processing for creating masks based on the artifact image (The image marked with a red box in (k)).

As the synthesized VMIs highly depend on the quality of the MBIs, the core idea of the proposed rMAR algorithm is to obtain the corrected MBIs for low‐energy VMI reconstruction. A series of VMIs ranging from 40 to 200 keV was acquired using a commercial dual‐energy spectral CT system. For selected VMIs (e.g., 70, 80, 100, 120, and 140 keV), a preliminary metal artifact reduction is applied, which will be described in detail later. Artifact images can be obtained by subtracting the preliminary corrected VMIs from the corresponding original VMIs, as illustrated in Figure 2(a–j). The extracted artifact images (Figure 2f–j) extracted from the VMIs sequence (Figure 2a–e) are analyzed to determine the optimal VMI (e.g., with minimal artifact) using specific evaluation criteria such as total variation (TV).^23^ As shown in Figure 2(l), the VMI at 100 keV exhibited the lowest TV value, and 100 keV was thus identified as the optimal energy level. The TV‐based normalized cost function was calculated as follows:

(2) $$C_{norm}\left(n\right)=\frac{C\left(n\right)}{\max\left(C\left(n\right)\right)},\;\;n\;=1,\;2,\;\cdots ,\;N$$

where

(3) $$C\;\left(n\right)=\sum_{x}\sum_{y}\sqrt{{\left(f_{x+1,y}-f_{x,y}\right)}^{2}+{\left(f_{x,y+1}-f_{x,y}\right)}^{2}}\;\left(n\right)$$

where *n* represents the index of the input images, ranging from 1 to *N*. *N* is the total number of input images. C(n) denotes the cost function value for the *n*‐th input image. $f_{x,y}$ denotes the pixel value at the coordinate position (*x*, *y*) in the image f.

After identifying the VMI with the most severe metal artifacts, artifact and non‐artifact regions were segmented using an automatic masking method. The detailed workflow for obtaining artifact images and corresponding masks is depicted in Figure 2(k). Briefly, the VMI image with the most severe artifacts was selected as input. Threshold‐based segmentation was first applied to separate metal components from soft tissue. Subsequently, a Radon transform was performed on both images to generate projection data. The soft tissue projection was interpolated based on the metal projection to mitigate artifacts. A corrected image was then reconstructed from the modified soft tissue projection, following a procedure similar to previously published methods.^4^, ^9^ For the proposed algorithm, it was sufficient to extract regions with the most prominent artifacts rather than perform precise artifact correction. The aim is to localize artifact‐prone regions rapidly. Finally, the artifact image (highlighted by the red box in Figure 2k) was obtained by subtracting the corrected image from the input image.

Mask generation is based on the artifact image. The key step is the sorting and thresholding segmentation (STS), as shown on the right side of Figure 2(k). Each row of the artifact image is traversed, and the data within each row is sorted to obtain the median. Upper and lower boundaries were determined by adding and subtracting a constant value (10 HU was used) from the median, respectively. Areas lying outside the boundaries are identified as artifact regions. The localized approach was chosen instead of a global image‐based method because it is challenging to identify a universal threshold that could yield satisfactory results for different datasets. Finally, morphological operations such as dilation and erosion are applied to refine the results. An example of STS processing is illustrated in Figure 2(m), where the black line represents the data from the 200^th^ row of the image, and the red and blue lines represent the threshold boundaries. In Figure 2(k), the STS module is applied twice. The outputs of both STS processes are binary masks. The first STS aims to obtain the obvious artifact region from an artifact image (labeled as STS1). The second STS (referred to as STS2) is implemented to acquire a better artifact‐free mask. STS1 and STS2 follow the same processing steps. The difference is the structuring element used in the erosion step. STS1 employs a 4‐pixel element, while STS2 uses a 1‐pixel element. This is because the input images processed by STS1 typically exhibit stronger artifacts compared to the images processed by STS2.

**FIGURE 3:**
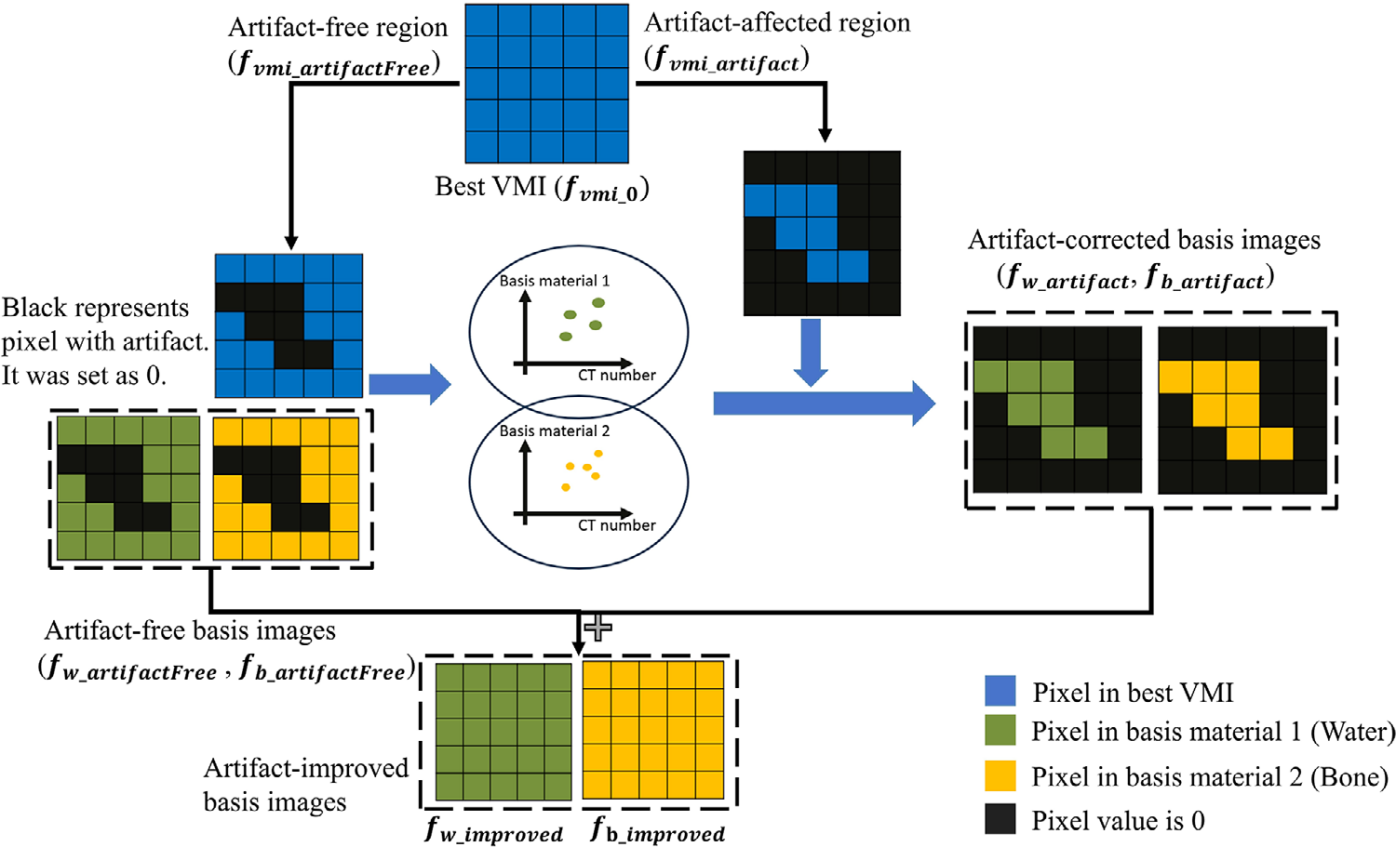
Scheme of the rMAR algorithm.

**FIGURE 4:**
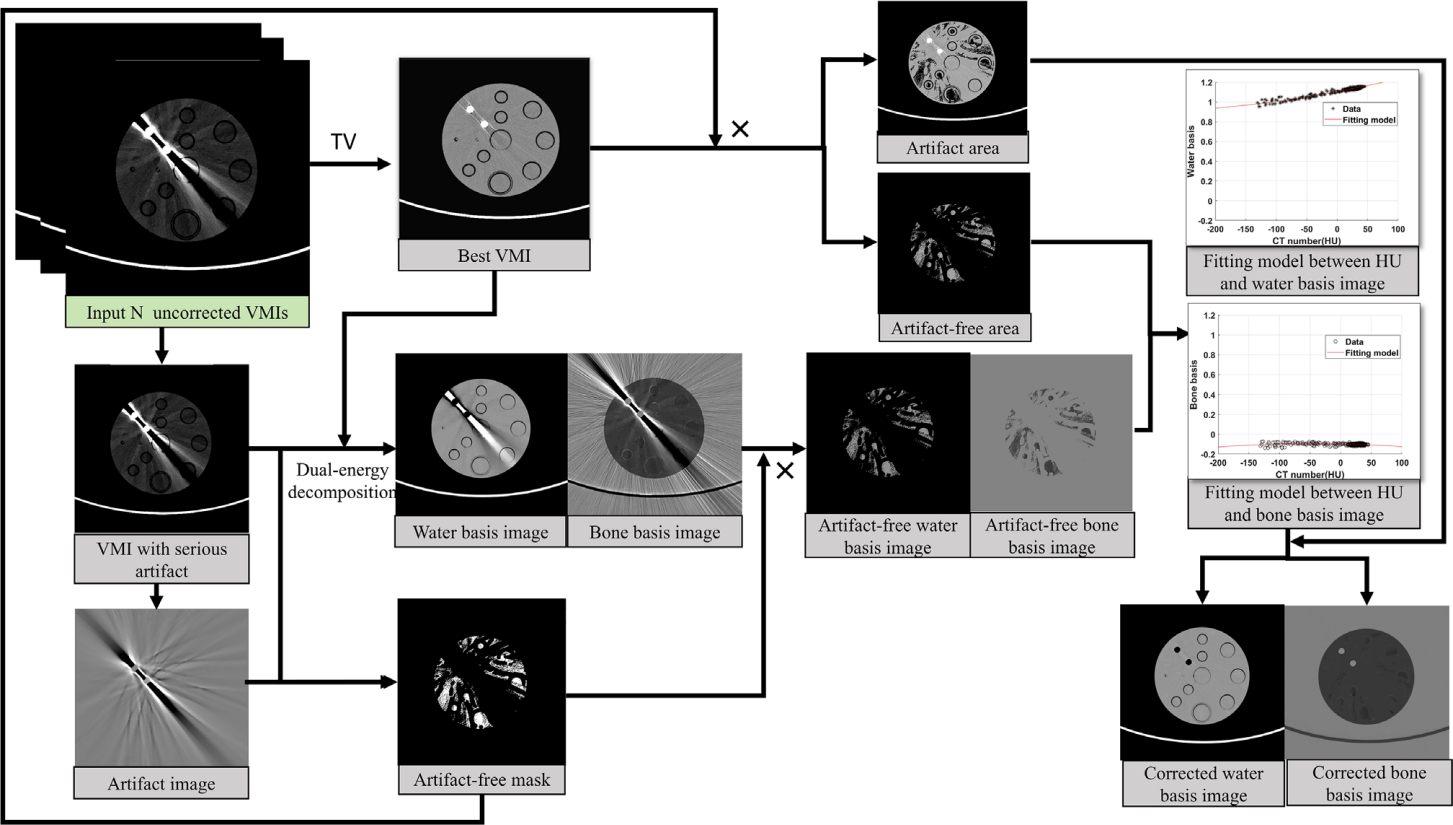
rMAR workflow.

Using the generated masks, the optimal VMI can be split into artifact‐free and artifact‐affected regions. An optimized mapping model between VMI and MBIs can be established using data from the artifact‐free region. This model is then applied to the artifact‐affected regions in VMI to correct the corresponding areas in the MBIs. The corrected MBIs are subsequently used to synthesize improved VMIs with reduced artifacts. This method is referred to as the regional model for metal artifact reduction (rMAR). The framework of the algorithm is shown in Figure 3. Figure 4 details its implementation using a specific case to enhance understanding of the methodology.

**FIGURE 5:**
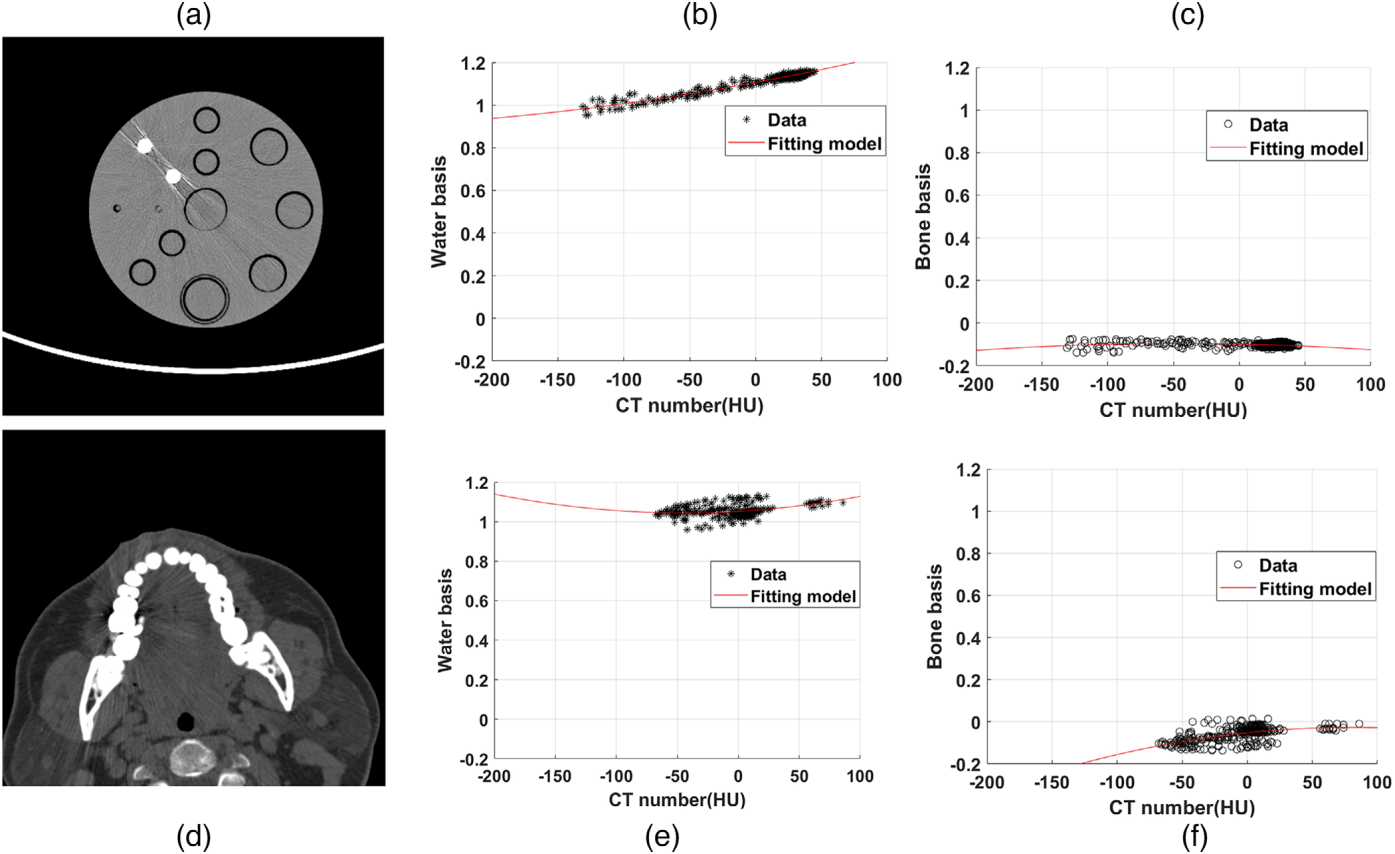
(a) The best VMI at 100 keV. Two 10 mm titanium rods are placed in the phantom. The window level and width were set to 0/200 HU. (b) The model based on the CT number and water basis of (a). (c) The model based on the CT number and bone basis of (a). (d) The best VMI of the patient 2 is at 140 keV. The window level and width were set to 200/700 HU. (e) CT number and water basis model from (d). (f) CT number and bone basis model from (d).

Figure 5 shows that the regional mapping model was developed using a quadratic polynomial fit, which yielded robust results. The fitting was performed in MATLAB, and the polynomial parameters vary as a function of the input data. The material basis maps represent physical density fractions. Notably, the bone material components appear negative in Figure 5(c, f). This can be attributed to the following factors. Bone and metal structures were excluded before the model construction. In the phantom image shown in Figure 5(a), no bone structure is present. The phantom material is composed of solid water rather than pure water, which leads to negative values in the bone basis image (Figure 5c). If the scanned object were consisted of pure water, the bone basis map would be zero. Similarly, in patient data shown in Figure 5(d), soft tissues are not composed solely of water. It also contains fat and other biological components.

An itemized description of the new method is described as follows:

1. Derive MBIs from the VMI sequence. In the study, the water‐based image ($f_{w})$ and bone basis image ($f_{b}$) were chosen.
2. Identify the best VMI, denoted as $f_{vmi\_0}$.
3. Generate the artifact mask image ($f_{M1}$), and define the artifact‐free mask image as $f_{M0}=1-\;f_{M1}$.
4. By applying $f_{M0}$ to $f_{w}$, $f_{b}$, and $f_{vmi\_0}$, the corresponding artifact‐free images were generated, denoted as $f_{w\_artifactFree}$, $f_{b\_artifactFree}$, and $f_{vmi\_artifactFree}$.
5. Establish a region‐based mapping model by optimizing the paired data of ($f_{vmi\_artifactFree}$, $f_{w\_artifactFree}$) and ($f_{vmi\_artifactFree}$, $f_{b\_artifactFree}$), as illustrated in Figures 5(a–c) for phantom imaging and Figures 5(d–f) for dental imaging.
6. The mask $f_{M1}$ was applied to $\;f_{vmi\_0}$ to obtain the artifact‐affected image $f_{vmi\_artifact}$, which was then mapped to the corresponding MBIs $f_{w\_artifact}$ and $f_{b\_artifact}$ using the regional mapping model from step 5.
7. The artifact‐improved basis images were synthesized as follows:
(4) $$f_{w\_\mathrm{impr}\mathrm{oved}}=f_{w\_\mathrm{arti}\mathrm{fact}\mathrm{Free}}+f_{w\_\mathrm{arti}\mathrm{fact}}$$
(5) $$f_{b\_\mathrm{impr}\mathrm{oved}}=f_{b\_\mathrm{arti}\mathrm{fact}\mathrm{Free}}+f_{b\_\mathrm{arti}\mathrm{fact}}$$
8. Based on artifact‐improved MBIs, a new set of VMIs can be synthesized.

## 3 RESULTS

### 3.1 Phantom evaluation

#### 3.1.1 Qualitative analysis

**FIGURE 6:**
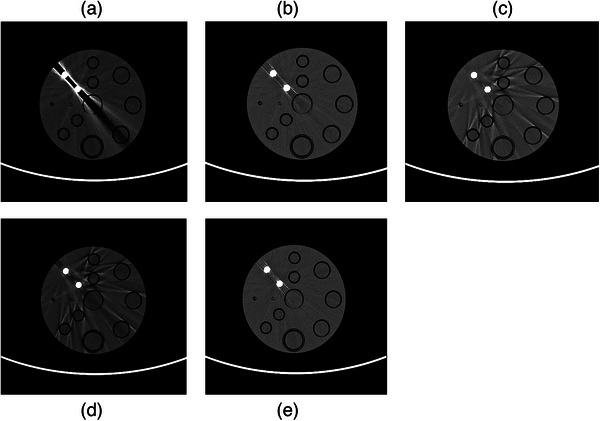
Images of a 200 mm solid water phantom with two 10 mm Ti rods. (a) VMI at 70 keV. (b) VMI at 100 keV. (c) VMI + OMAR at 100 keV. (d) VMI + OMAR at 70 keV. (e) VMI + rMAR at 70 keV. The window level and width were set to 0/200 HU.

Figure 6(a) shows the VMI at 70 keV, while Figure 6 (b) demonstrates the optimal VMI at 100 keV, which provides superior metal artifact reduction in this case. Figure 6 (c, d) presents the results of VMI combined with OMAR (VMI + OMAR) at 100 and 70 keV, respectively. Figure 6 (e) illustrates the outcome of the VMI combined with the rMAR (VMI + rMAR) method at 70 keV. A comparison between Figure 6(a) and Figure 6(b) demonstrates that the higher energy image achieves better metal artifact suppression. Furthermore, OMAR introduces additional artifacts as seen in Figure 6(c, d), whereas the proposed rMAR method effectively reduces the metal artifacts without introducing new ones, as shown in Figure 6 (e).

#### 3.1.2 Quantitative evaluation

**FIGURE 7:**
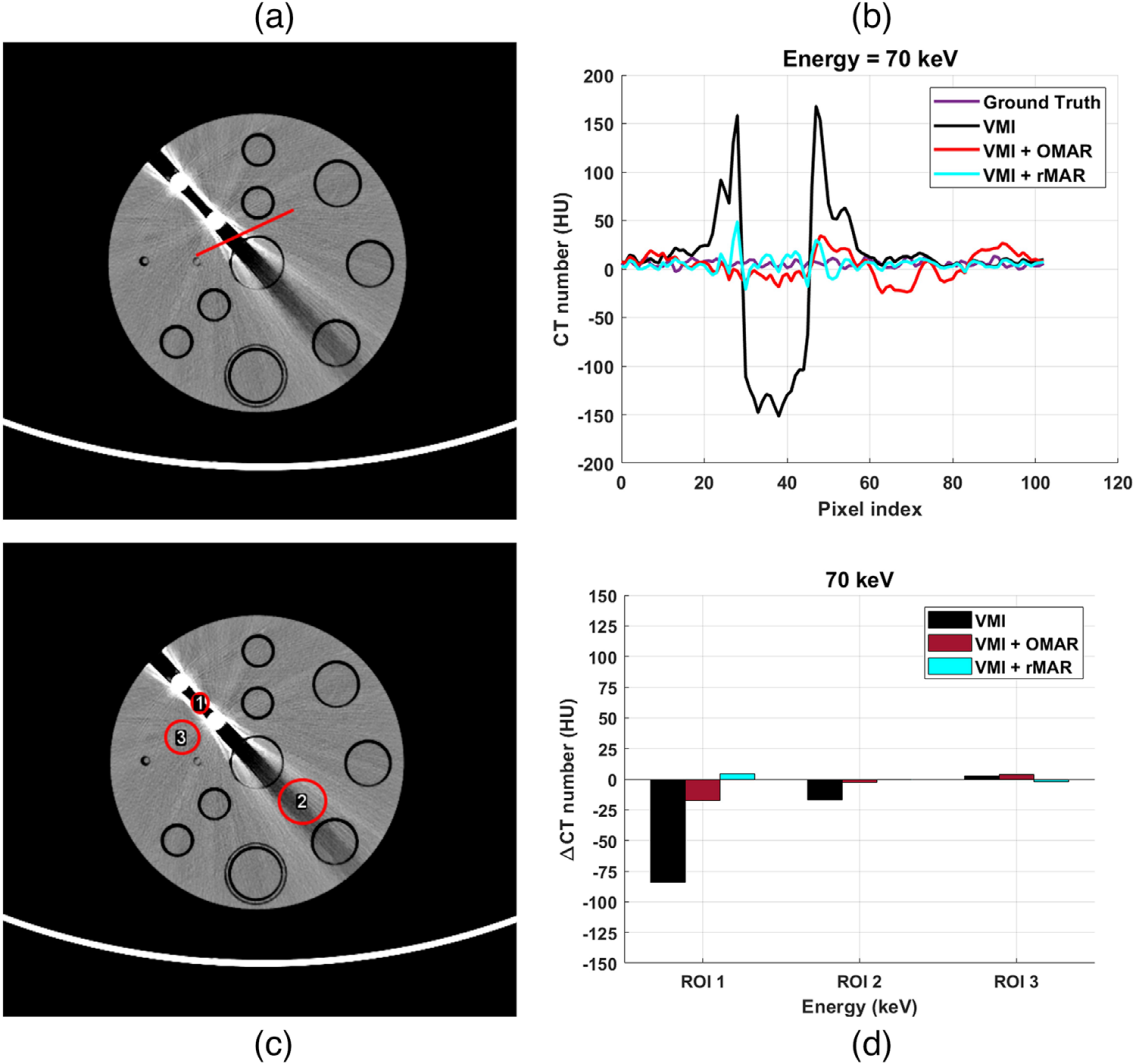
Quantitative evaluation. (a)The position of the selected lines in the phantom. (b) The profiles from imaging. (c)The position of the selected ROIs in the phantom. (d) The ΔCT number of imaging. The window level and width were set to 0/200 HU.

Figure 7(a) illustrates the locations of the selected line used for profile evaluation. The ground truth corresponds to VMI with all water insertions. As shown in Figure 7(b), the proposed rMAR method yields an image with fewer metal artifacts compared to those processed with OMAR. The ground truth profile exhibits an approximate value of 0 Hounsfield Units (HU), whereas the uncorrected profile (black line) demonstrates a significant deviation from this reference standard (purple line). However, both OMAR and rMAR effectively mitigate these artifacts, resulting in profiles that closely align with the ground truth.

Figures 7(c, d) present quantitative comparisons based on the mean CT value in three regions of interest (ROIs). The ΔCT is defined as the difference between the mean CT value (CT) and a reference value ($CT_{ref}$) within a given ROI:

(6) $$\Delta CT=CT-CT_{ref}$$

A smaller ΔCT value indicates a more accurate correction result. The uncorrected VMI exhibits a ΔCT value of about 85 HU. Quantitative analysis revealed that OMAR was less effective than rMAR in ROI 1, with a ΔCT of approximately 15 HU. Overall, rMAR outperformed OMAR in three evaluated ROIs.

### 3.2 Patient validation

**FIGURE 8:**
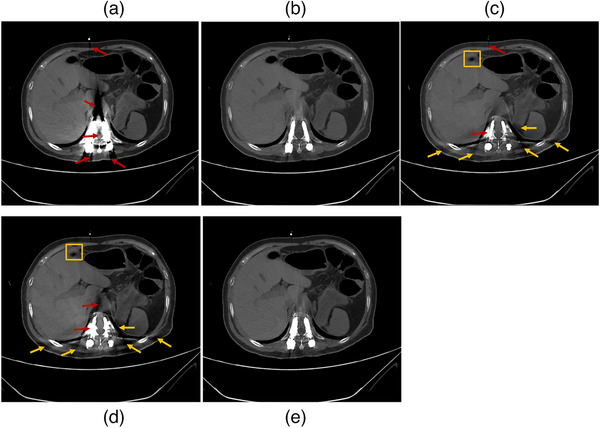
Postoperative VMIs of patient 1. (a) VMI at 70 keV. (b) VMI at 140 keV. (c) VMI + OMAR at 140 keV. (d) VMI + OMAR at 70 keV. (e) VMI + rMAR at 70 keV. The window level and width were set to 200/700 HU.

**FIGURE 9:**
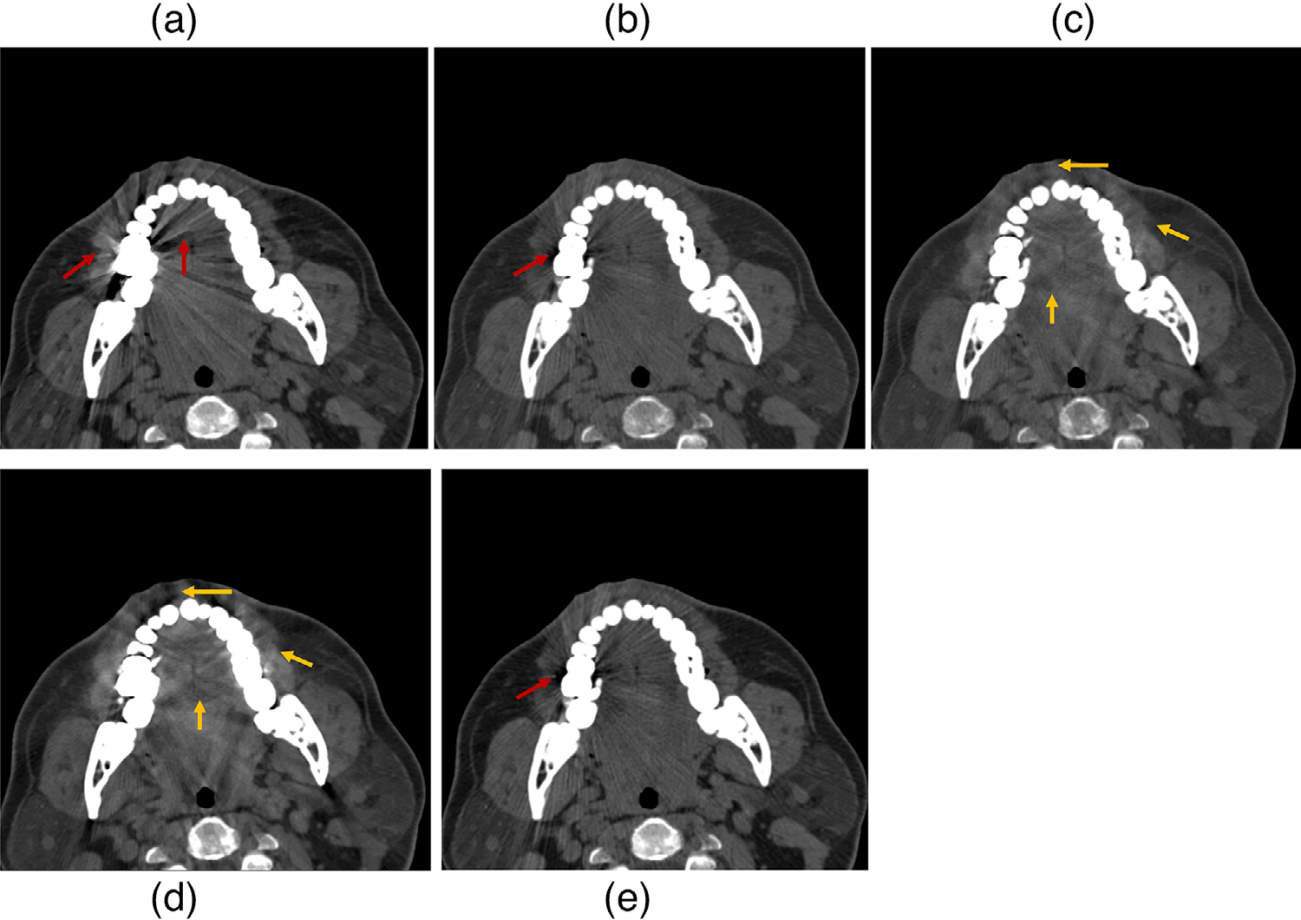
Postoperative VMIs of patient 2. (a) VMI at 70 keV. (b) VMI at 140 keV. (c) VMI + OMAR at 140 keV. (d) VMI + OMAR at 70 keV. (e) VMI + rMAR at 70 keV. The window level and width were set to 200/700 HU.

**FIGURE 10:**
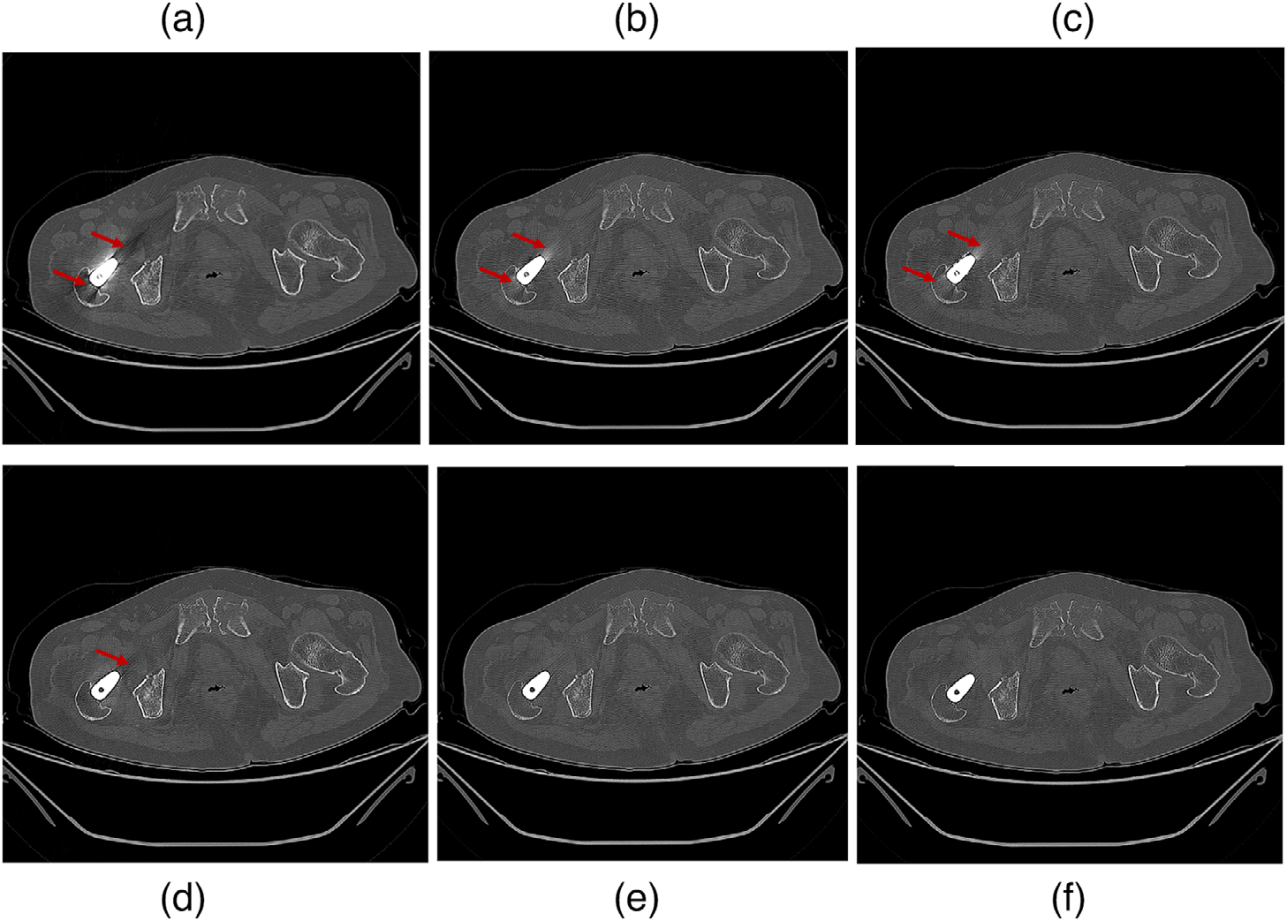
Postoperative VMIs of patient 3′ hip prosthesis implant. (a) VMI at 70 keV. (b) VMI at 140 keV. (c) VMI+ rMAR at 70 keV. (d) VMI + OMAR at 70 keV. (e) VMI + OMAR at 140 keV. (f) VMI + OMAR + rMAR at 70 keV. The window level and width were set to 500/2500 HU.

Data from three patients is presented in Figures 8, 9, 10. Four different image processing methods were applied: VMI, VMI + OMAR, VMI + rMAR, and VMI + OMAR + rMAR. Red arrows indicate metal artifacts present in the original VMIs. Yellow arrows denote new artifacts introduced by the correction algorithm (OMAR or rMAR). Yellow boxes highlight regions where tissue structures have been altered due to the application of artifact reduction algorithms.

Figure 8 shows the VMIs acquired from a patient with pedicle screw fixation in the thoracic vertebra. Figure 8(a, b) displays VMIs at 70 and 140 keV, respectively. Figure 8(c–e) shows the results of VMI + OMAR at 140 keV, VMI + OMAR at 70 keV, and VMI + rMAR at 70 keV, respectively. Low‐energy VMIs (Figure 8a) exhibit substantial metal artifacts, especially near the metal. Metal boundaries become indistinguishable from adjacent bone and soft tissue areas. High‐energy VMI (Figure 8b) reduces artifacts effectively. The OMAR algorithm introduces new artifacts in both high‐ and low‐energy VMIs (Figure 8c, d). Moreover, it tends to oversmooth surrounding soft tissue, resulting in visible alterations to anatomical structure (highlighted by the yellow box). In contrast, the rMAR (Figure 8e) demonstrates more effective artifact reduction with better preservation of tissue detail.

Figure 9 shows patient data with dental fillings. The results of VMI combined with OMAR (Figure 9c, d) show a tendency toward over‐smoothing of soft tissue. In contrast, the VMI + rMAR method effectively reduces the metal artifacts in low‐energy VMI (Figure 9e) while better preserving anatomical details. Although some residual artifacts remain, the clarity of the soft tissue structure is maintained.

Figure 10 depicts a case with a hip prosthesis, which has a regular and symmetric shape. Consequently, both high‐ and low‐energy VMIs exhibit mild metal artifacts. In addition, because there is a residual metal artifact in the high‐energy VMI image (Figure 10b), the VMI + rMAR at 70 keV also retains artifacts (Figure 10c). The result in Figure 10(c) demonstrates superior performance compared to Figure 10(a). The rMAR algorithm can be further applied to MAR‐processed images for enhanced artifact correction. A combined approach using VMI + OMAR + rMAR (Figure 10f) further enhances image quality by leveraging the advantages of both correction methods. In summary, the proposed rMAR algorithm is flexible and can be applied either before or after MAR processing, depending on the performance of the MAR algorithm in a given case.

## 4 DISCUSSIONS

When metal artifacts are present, VMIs at lower energy levels demonstrate more severe metal artifacts than at higher energy levels. Although commercial MAR techniques can reduce such artifacts, they may introduce new artifacts. In this study, we proposed a regional‐mapping‐based metal artifact reduction in the image domain, specifically designed to improve low‐energy VMIs when high‐energy VMIs demonstrate sufficient image quality. After establishing a model between the optimal VMI and corresponding MBIs in artifact‐free regions, the model was subsequently employed for correcting artifact‐contaminated regions in the MBIs. The corrected MBIs can be used to synthesize improved low‐energy VMIs without introducing new artifacts. Validation was conducted using both phantom and clinical patient data. To further evaluate the generalizability of the rMAR method, future studies should involve datasets acquired from different CT scanners.

Interestingly, the optimal VMI energy varied across test cases: 100 keV for the phantom study and 140 keV for patient data. Both the patient and phantom data were acquired using the same Philips CT scanner. Although all patient scans in this study were performed using a 140 kV protocol, it does not mean that 140 keV is universally optimal for all cases. Previous studies have shown that the optimal VMI energy level for metal artifact reduction can vary significantly depending on the type and location of the metal implant, with effective energy levels generally ranging from 95 and 150 keV.^6^, ^22^ Moreover, the artifact reduction performance is not strictly proportional to the energy levels, further indicating that the optimal energy must be determined on a case‐by‐case basis. Therefore, the proposed rMAR algorithm requires individual selection of the optimal VMI prior to application. In this study, the optimal VMI selection process was implemented in a fully automated manner.

The robustness of the proposed rMAR algorithm should be further demonstrated in scenarios involving multiple metallic implants. Although such cases generally present severe artifacts, satisfactory correction can still be achieved provided the input VMI was of reasonable quality. The approach works effectively with either native or OMAR‐corrected high‐energy VMIs, and its design in the image domain makes it inherently compatible with all spectral CT systems.

In existing literature, the performance of MAR algorithms is typically evaluated using both quantitative and qualitative methods. Quantitative assessments often include the accuracy of HU values and image noise measurements.^3^, ^11^, ^16^ The assessment of clinical image quality commonly relies on qualitative evaluations by trained radiologists, especially in cases where definitive ground truth standards are absent.^4^, ^9^ In addition, statistical analysis methods are widely used to compare algorithm performance across studies.^24^

Direct quantitative analysis is currently not suitable for the proposed model, because the current model lacks a direct and interpretable mapping between the model coefficients and the HU values. In phantom studies, where ground‐truth images are available, we performed quantitative evaluations by measuring HU differences. In contrast, patient scans generally lack metal‐free references, necessitating qualitative assessments based on visual inspection. Future iterations of the model should aim to provide more structured and quantifiable mappings to facilitate robust evaluation.

Notably, rMAR is targeted at low‐energy VMIs. Low‐energy VMIs provide superior contrast but are severely impaired by metal artifacts in many cases. High‐energy VMIs, while less prone to artifacts, are often diagnostically sufficient without correction. Although this study utilized data from the Philips scanner, it is generally observed that commercial MAR algorithms tend to perform poorly in low‐energy VMIs. For instance, the lack of improvement in Figure 8(c) may be attributed to the complex geometry of the pedicle screw, which limits the effectiveness of the OMAR algorithm.^23^ Nevertheless, the proposed rMAR achieved promising results across both phantom and clinical datasets. This justifies the specific application of rMAR to low‐energy VMIs.

The aim of the proposed algorithm is to use high‐energy VMI for correcting low‐energy VMI. High‐energy VMIs serve as the foundation for model construction, so the algorithm cannot further improve the high‐energy VMIs. Nevertheless, the algorithm's reliance on the best VMI introduces inherent limitations. In cases of severe photon starvation—such as in hip prosthesis imaging—none of the available energy levels may yield acceptable VMI quality, which compromises the effectiveness of the proposed algorithm. Integrating existing MAR methods before applying rMAR (e.g., VMI + OMAR + rMAR) may improve the initial quality. In addition, if MAR unfortunately causes values in the artifact‐affected region in the best VMI to deviate from the ground truth, rMAR results will also be impacted by this error. The magnitude of the induced error depends on the initial image error and the accuracy of the model. Furthermore, the use of high‐energy VMI for model construction may limit the retention of subtle soft tissue contrast in corrected images. However, low‐energy VMIs generally suffer from serious artifacts if there are metal implants, causing the images to lose their clinical diagnostic value. Therefore, in clinical practice, this trade‐off is often acceptable if the anatomical structures are preserved and the overall artifact burden is significantly reduced.

The proposed method cannot be directly applied to conventional polychromatic images, such as 140 or 70 kV. Because the core of the algorithm is to build a model based on the non‐artifact regions of high‐energy VMIs and basis images. The principles may be adaptable to polychromatic imaging with appropriate modifications in the future. The corrected MBIs produced by rMAR also enable the generation of other clinically useful outputs, such as material density maps, virtual non‐contrast images, iodine maps, electron density images, and effective atomic number images.

While current validation focused on two‐dimensional axial slices, the framework can be extended to three‐dimensional volumetric datasets to enhance spatial consistency. In addition, the best VMI selection currently occurs within a single slice. Future improvement may incorporate adjacent slices with fewer artifacts to enhance model robustness.

## 5 CONCLUSIONS

This study demonstrates the effectiveness of the proposed rMAR algorithm for low‐energy VMIs in the image domain. Both phantom and clinical data acquired from a commercial spectral CT scanner were used for validation. The rMAR approach robustly suppresses metal artifacts while maintaining anatomical details.

## CONFLICT OF INTEREST STATEMENT

The authors declare no conflicts of interest.
